# Plasma biomarker ORAI1 as a dual prognostic value for survival and postoperative quality of life in glioma patients

**DOI:** 10.1038/s41598-025-34228-4

**Published:** 2025-12-30

**Authors:** Zeyu Zhang, Yu Xiang, Ying Zhang, Hui Li, Yun Cheng

**Affiliations:** 1 Department of Anesthesiology, Hangzhou Institute of Medicine (HIM), Zhejiang Cancer Hospital, Chinese Academy of Sciences, Hangzhou, 310022 Zhejiang China; 2 Department of Neurology, Shanxi Provincial People’s Hospital, Taiyuan, 030001 China; 3 Department of Anesthesiology, Zhejiang Cancer Hospital, No. 1 Banshandong Road, Hangzhou, 310022 Zhejiang P.R. China

**Keywords:** Glioma, Prognosis, Biomarker, Biomarkers, Cancer, Oncology

## Abstract

Gliomas represent one of the most common and highly malignant primary brain tumors, with overall prognosis remaining unsatisfactory to date. Although certain molecular biomarkers have been identified to stratify patients based on survival outcomes, non-invasive blood biomarkers capable of simultaneously predicting both survival prognosis and postoperative quality of life (QoL) are still lacking. This study provides substantial evidence for the potential of ORAI1 as a non-invasive biomarker across diverse ethnic clinical cohorts. This investigation employed a translational medicine-oriented exploratory approach, incorporating 309 glioma patients from the Chinese Glioma Genome Atlas (CGGA) and 563 patients from The Cancer Genome Atlas (TCGA). Additionally, preoperative plasma samples from 50 glioma patients were analyzed by ELISA, alongside postoperative QoL assessments within three days after surgery. The correlations between ORAI1 gene expression and patients’ survival outcomes as well as postoperative QoL were evaluated, with further exploration of potential underlying biological mechanisms. High ORAI1 expression was identified as an independent adverse prognostic factor in glioma patients, significantly enriched in those with higher WHO grades (III–IV). Patients in the high ORAI1 expression group exhibited increased postoperative pain scores and poorer sleep quality. Notably, ORAI1 expression was absent or minimal in glioma tissues but markedly elevated in patients’ preoperative plasma. Functional enrichment analyses including Gene Ontology (GO), Kyoto Encyclopedia of Genes and Genomes (KEGG), and Gene Set Variation Analysis (GSVA) indicated the potential utility of ORAI1 as a peripheral blood biomarker. ORAI1 serves not only as an independent predictor of survival prognosis but also reflects postoperative QoL, representing a promising dual biomarker. The discordant expression of ORAI1 in tumor tissue versus plasma suggests a unique role in tumor–host system interactions. These findings suggest new directions for precise prognostic evaluation and personalized perioperative management in glioma patients.

**Supplementary Information:**

The online version contains supplementary material available at 10.1038/s41598-025-34228-4.

## Introduction

Gliomas, particularly glioblastoma (GBM), represent the most common and highly aggressive primary malignant tumors of the adult central nervous system, accounting for approximately 60–80% of all malignant brain tumors^1^. Despite current treatment modalities encompassing maximal safe surgical resection, radiotherapy, chemotherapy, and emerging targeted therapies, the prognosis for high-grade glioma patients remains dismal, with median overall survival typically not exceeding 15 months^2,3^. Furthermore, postoperative neurological deficits and treatment-related complications—such as radiation-induced brain injury and chemotherapy-associated cognitive impairment—significantly compromise patients’ postoperative QoL, representing critical determinants of long-term recovery^4^.

To improve clinical classification and therapeutic decision-making, molecular biomarkers including isocitrate dehydrogenase (IDH) mutation, 1p/19q codeletion, and O6-methylguanine-DNA methyltransferase (MGMT) promoter methylation have been widely incorporated into glioma classification and prognostic evaluation, and were integrated as core diagnostic criteria in the 2021 WHO classification of central nervous system tumors^5^. The application of these markers has substantially enhanced risk stratification for survival and individualized treatment planning^6^. However, these molecular assays depend on invasive tissue sampling via surgery or biopsy, which entails high costs, procedural risks, and precludes dynamic monitoring. Moreover, existing tissue biomarkers primarily focus on tumor molecular subtyping, lacking predictive capacity for subjective clinical outcomes such as postoperative pain and sleep quality^7^. In addition, non-coding RNAs have emerged as critical regulators of glioma angiogenesis and tumor microenvironment, highlighting the potential of circulating molecular markers as minimally invasive biomarkers for glioma detection and monitoring^8^.

Recently, liquid biopsy-based non-invasive biomarkers have gained increasing attention, particularly circulating tumor DNA, exosomal proteins, and microRNAs detectable in peripheral blood, emerging as promising alternative diagnostic tools^9^. Compared with traditional tissue specimens, blood-based biomarkers offer advantages of safety, repeatability, and real-time dynamic monitoring, which are especially suitable for longitudinal tumor surveillance and functional status evaluation after surgery^10^. Moreover, targeted therapies such as IDH1/2 inhibitors for low-grade gliomas demonstrate the clinical importance of identifying molecular alterations, underscoring the value of blood-based markers for non-invasive monitoring and guiding precision treatment^11^. Among these, circulating proteins hold broad clinical translational potential due to their relatively simple detection, cost-effectiveness, and ability to reflect complex tumor–host systemic interactions^12^.

ORAI1, a key component of the store-operated calcium entry (SOCE) channel, was originally identified in immune cells and shown to regulate calcium signaling^13^. In recent years, aberrant expression of ORAI1 has been reported in various solid tumors, where it participates in regulating tumor initiation, proliferation, invasion, and metastasis^14^. Studies have demonstrated ORAI1 overexpression in breast, pancreatic, and lung cancers, closely associated with malignant phenotypes such as enhanced proliferation, migration, and therapy resistance^15^. For instance, Gu et al. reported that the interaction between ORAI1 and nucleolin promotes breast cancer cell proliferation^15^, while Pan et al. confirmed that ORAI1 upregulation facilitates cervical cancer progression via cell cycle modulation^14^.

However, systematic investigations of ORAI1 in gliomas remain lacking, especially regarding its expression in peripheral blood and its relationship with patient prognosis and postoperative QoL. Therefore, this study aims to evaluate the potential clinical value of ORAI1 in glioma, focusing on its predictive utility as a non-invasive blood biomarker.

We integrated transcriptomic data from the TCGA and CGGA databases, and prospectively collected plasma samples from 50 glioma patients at our center to quantify ORAI1 levels by ELISA. We concurrently assessed associations between ORAI1 expression, tumor grade, patient survival, and QoL indicators, including sleep and pain scores. Through survival analyses, ROC curves, and GSVA pathway enrichment, we further elucidated key biological pathways potentially involving ORAI1, emphasizing its roles in calcium homeostasis, endoplasmic reticulum stress, and extracellular vesicle trafficking.

In summary, this study centers on ORAI1 as a novel blood-based biomarker candidate, aiming to assess its dual predictive value for tumor biological behavior and postoperative functional outcomes. Our findings may provide new theoretical support for the clinical translation of liquid biopsy in glioma management.

## Materials and methods

### Patients and samples

Gene expression profiles and clinical data of glioma patients were obtained from the CGGA (https://www.cgga.org.cn/) and TCGA (https://portal.gdc.cancer.gov/) databases^14^. For the independent cohort, peripheral blood plasma samples and clinical information from 50 glioma patients were collected following approval by the Institutional Review Board (IRB) of Zhejiang Cancer Hospital. Inclusion criteria were: age ≥ 18 years, no prior radiotherapy or chemotherapy, adequate preoperative cognitive and communication abilities, and provision of informed consent. Exclusion criteria included: pre-existing or concurrent conditions that could affect quality of life (such as chronic pain, chronic insomnia, or other severe systemic diseases), severe cardiac, hepatic, or renal dysfunction, concomitant malignancies, or pregnancy. Molecular pathological analyses—including detection of IDH mutations, 1p/19q codeletion, and MGMT promoter methylation—were performed at the Molecular Pathology Diagnostic Center of Zhejiang Cancer Hospital. Postoperative follow-up was conducted for three consecutive days, during which relevant clinical data were recorded.

### Analyze ORAI1 expression in glioma

The GEPIA database (http://gepia2.cancer-pku.cn/#analysis) was utilized to compare ORAI1 expression between human cancers and matched normal tissues. Additionally, the Human Protein Atlas (HPA) database (https://www.proteinatlas.org/) was employed to explore ORAI1 expression in GBM and lower-grade glioma (LGG).

### Functional enrichment analysis

The genes most relevant to ORAI1, or characteristic gene lists from specific cell clusters, were submitted to the Database for Annotation, Visualization, and Integrated Discovery (DAVID, version 2023q4; https://david.ncifcrf.gov/). Official gene symbols were selected as identifiers, with Homo sapiens specified as the species. GO (http://geneontology.org/) and KEGG (www.kegg.jp/feedback/copyright.html) pathway enrichment analyses were performed. The study presents the top six enriched terms ranked by ascending p-values (*p* < 0.05)^16^.

### Gene set variation analysis

Gene lists related to immune processes were obtained from the AmiGO 2 portal (http://amigo.geneontology.org/amigo)^16^. Functional enrichment scores for each GBM sample were calculated using the specified R package under default parameters. Heatmaps of the enrichment results were generated using the base R heatmap() function (R version 4.5.1). Pearson correlation analysis was performed to evaluate the associations between ORAI1 expression and individual pathways as well as related genes.

### Clinical cohort and plasma sampling

A prospective cohort of 50 glioma patients who underwent surgery at Zhejiang Cancer Hospital between January 2023 and April 2024 was enrolled. Inclusion criteria were: age ≥ 18 years, histopathologically confirmed glioma, and no prior radiotherapy or chemotherapy before surgery. Five milliliters of peripheral blood were collected preoperatively and centrifuged at 3000 rpm for 10 min to separate plasma, which was then stored at − 80 °C. Plasma ORAI1 concentrations were quantified using a commercially available human ORAI1 ELISA kit (Wuhan Fine Biotech Co., Ltd., Wuhan, China; Cat# EH10774) according to the manufacturer’s instructions. The assay had a detection range of 0.156–10 ng/mL, and both intra- and inter-assay coefficients of variation were less than 10%, ensuring assay reliability.

### Postoperative quality of life assessment

Within 72 h after surgery, early postoperative quality of life was assessed, focusing on pain and sleep quality, which were evaluated using the Numeric Rating Scale (NRS) and the Pittsburgh Sleep Quality Index (PSQI), respectively.

### Statistical analysis

Statistical analyses were performed using R software (version 4.5.1; https://www.r-project.org/) and GraphPad Prism 10.0 (https://www.graphpad.com/). Univariate and multivariate Cox proportional hazards regression analyses were conducted to identify prognostic factors. Kaplan–Meier survival curves were compared using the log-rank test. Receiver operating characteristic (ROC) curves were employed to assess predictive performance. Group comparisons were performed using Student’s t-test or the Mann–Whitney U test as appropriate. A two-sided p-value < 0.05 was considered statistically significant.

## Results

### Enrichment of ORAI1 in neurogliomas harboring molecular markers predictive of malignancy

**Fig. 1:**
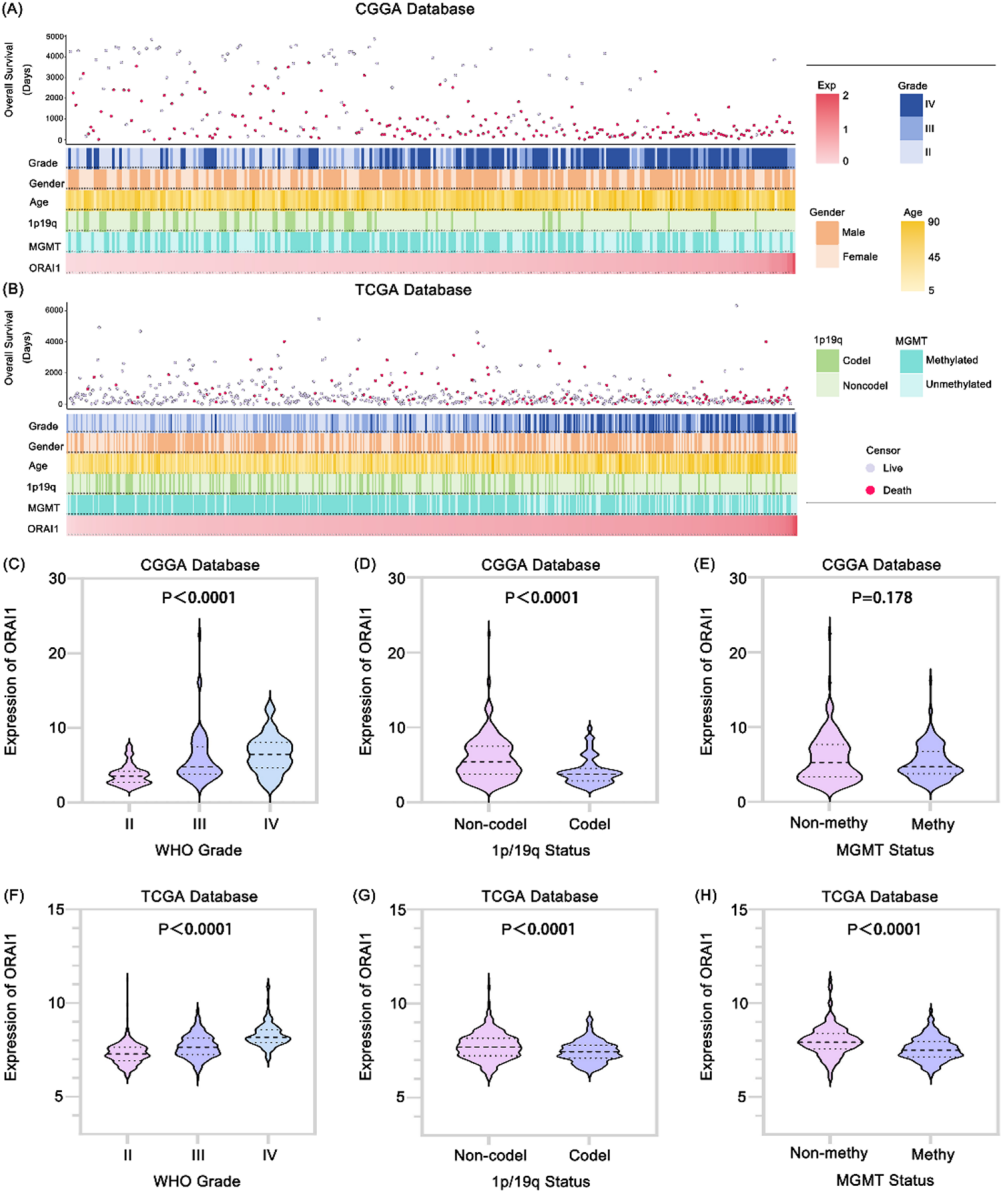
Association between ORAI1 expression and clinicopathological features in glioma. (**A**,** B**) Overview of ORAI1-associated clinicopathological characteristics in glioma from the CGGA (A) and TCGA (**B**) databases. (**C**,** F**) ORAI1 expression is significantly elevated in high-grade gliomas in both CGGA and TCGA cohorts, as determined by one-way ANOVA. (**D**,** G**) In both datasets, ORAI1 expression is higher in gliomas without 1p/19q codeletion; differences were assessed using unpaired t-tests. (**E**,** H**) ORAI1 expression is elevated in gliomas with unmethylated MGMT promoters. This difference reached statistical significance in the TCGA cohort but not in the CGGA cohort (unpaired t-test).

Patients with differing ORAI1 expression levels exhibited distinct clinical and pathological characteristics[Fig. 1A, B]. Variables including ORAI1 expression, MGMT promoter methylation status, 1p/19q codeletion status, WHO grade, sex, and age showed asymmetric distributions in both the CGGA and TCGA datasets[Table 1]. Comparative analyses were performed across these subgroups. In the CGGA database, ORAI1 was highly enriched in high-grade neurogliomas and samples lacking 1p/19q codeletion[Fig. 1C, D]. These findings were corroborated in the TCGA dataset[Fig. 1F, G]. Specifically, within the TCGA cohort, ORAI1 expression was significantly elevated in samples without MGMT promoter methylation[Fig. 1H]. A similar trend was observed in the CGGA dataset, although the difference did not reach statistical significance[Fig. 1E]. Collectively, these results suggest that neurogliomas with higher malignancy are characterized by elevated ORAI1 expression.

**Table 1:**
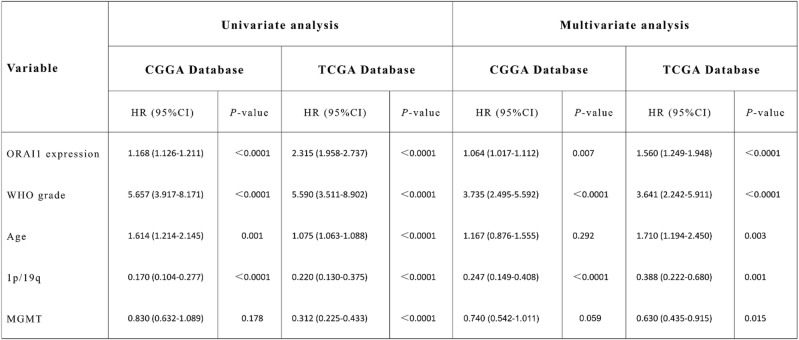
Univariate and multivariate Cox regression analyses for OS in CGGA and TCGA cohorts.

### High ORAI1 expression is associated with poor prognosis in glioma patients

**Fig. 2:**
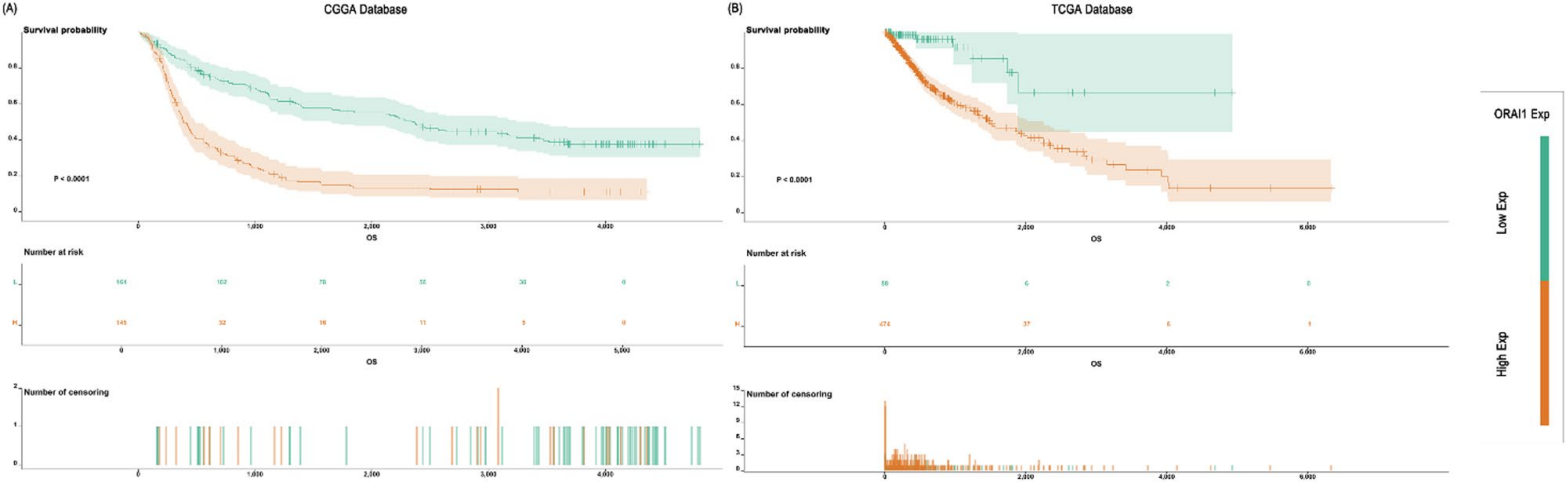
High ORAI1 expression predicts poor overall survival in glioma patients. (**A**,** B**) Kaplan–Meier survival curves based on the CGGA (**A**) and TCGA (**B**) cohorts show that patients with high ORAI1 expression have significantly shorter overall survival compared to those with low expression levels.

To investigate the relationship between ORAI1 expression and survival outcomes in glioma patients, Kaplan–Meier survival analyses were performed independently in the CGGA and TCGA cohorts. Patients were stratified into high and low expression groups based on the median ORAI1 expression level. Results demonstrated that, in the CGGA dataset, patients with low ORAI1 expression exhibited significantly longer overall survival (OS) compared to those with high expression (*p* < 0.01) [Fig. 2A]. Similarly, in the TCGA cohort, elevated ORAI1 expression was significantly associated with poorer survival outcomes (*p* < 0.01) [Fig. 2B]. These findings indicate that high ORAI1 expression is closely associated with poor patient survival.

### ORAI1 exhibits superior predictive performance in tumor grade classification

To further evaluate the potential value of ORAI1 in predicting glioma grade, receiver operating characteristic (ROC) curve analyses were conducted in both the CGGA and TCGA databases. The predictive performance of ORAI1 was compared with commonly used clinical molecular markers, including 1p/19q codeletion and MGMT promoter methylation status.

**Fig. 3:**
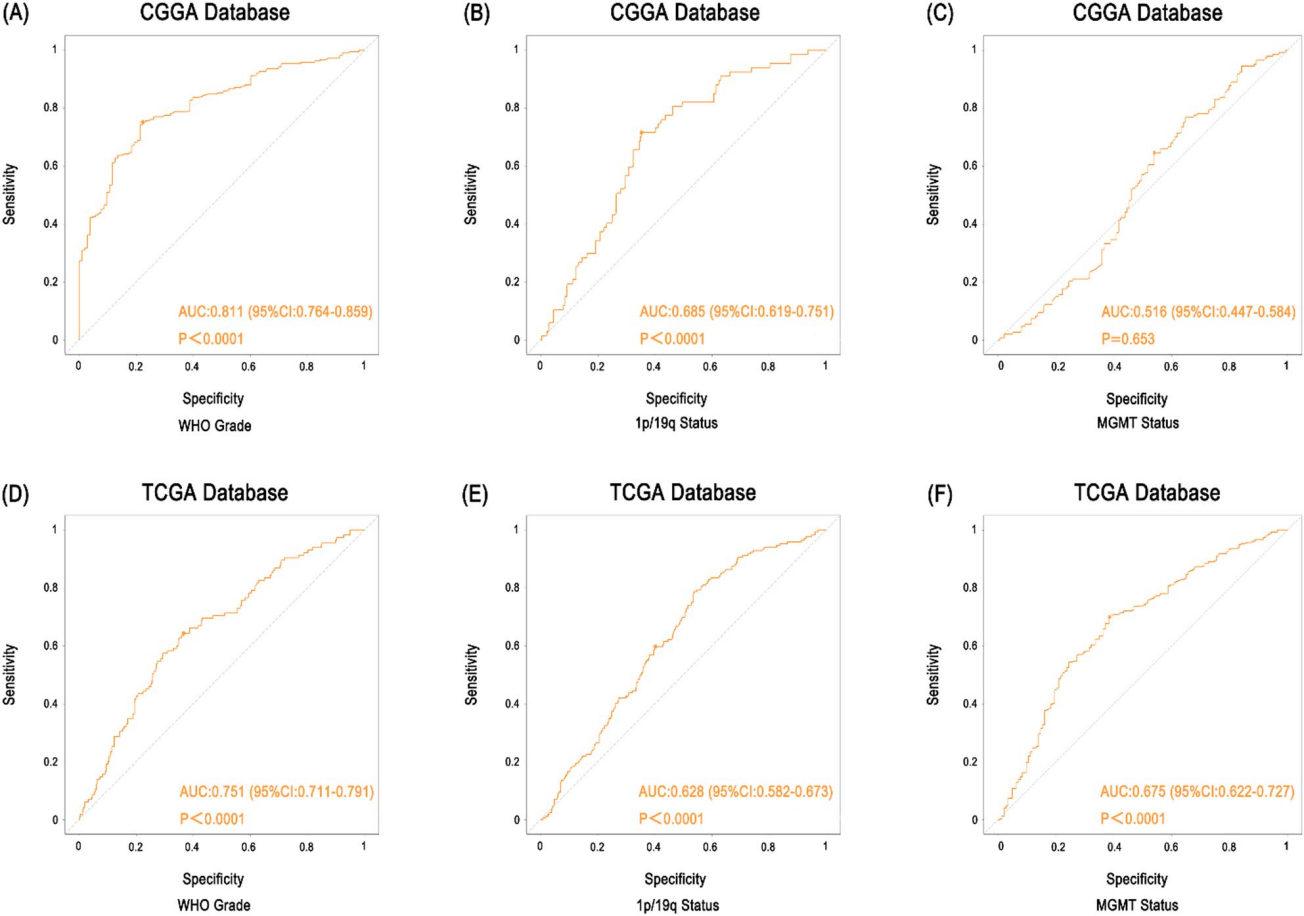
Diagnostic performance of ORAI1 in glioma molecular features. (**A–C**) In the CGGA cohort, ORAI1 distinguishes WHO grades well (AUC = 0.811, *P* < 0.001), moderately predicts 1p/19q codeletion (AUC = 0.685, *P* < 0.001), but not MGMT methylation (AUC = 0.516, *P* = 0.653). (**D–F**) In the TCGA cohort, ORAI1 shows good discrimination for WHO grades (AUC = 0.751, *P* < 0.001) and moderate prediction for 1p/19q codeletion (AUC = 0.628, *P* < 0.001) and MGMT methylation (AUC = 0.675, *P* < 0.001).

In the CGGA cohort, the area under the ROC curve (Area under the curve, AUC) for ORAI1 in predicting WHO tumor grade reached 0.811 (*p* < 0.001), significantly outperforming 1p/19q codeletion (AUC = 0.685, *p* < 0.001) and MGMT methylation (AUC = 0.516, *p* = 0.653) [Fig. 3A-C]. Similarly, in the TCGA dataset, ORAI1 demonstrated an AUC of 0.751 (*p* < 0.001) for WHO grade prediction, also surpassing 1p/19q (AUC = 0.628, *p* < 0.001) and MGMT (AUC = 0.675, *p* < 0.001) [Fig. 3D-F]. These statistically significant results across two independent cohorts indicate that ORAI1 possesses a stable and superior discriminative capability for tumor grade prediction.

### ORAI1 is hardly detectable in tumor tissues but is expressed in both peripheral blood and tumor tissue mRNA samples from glioma patients

**Fig. 4:**
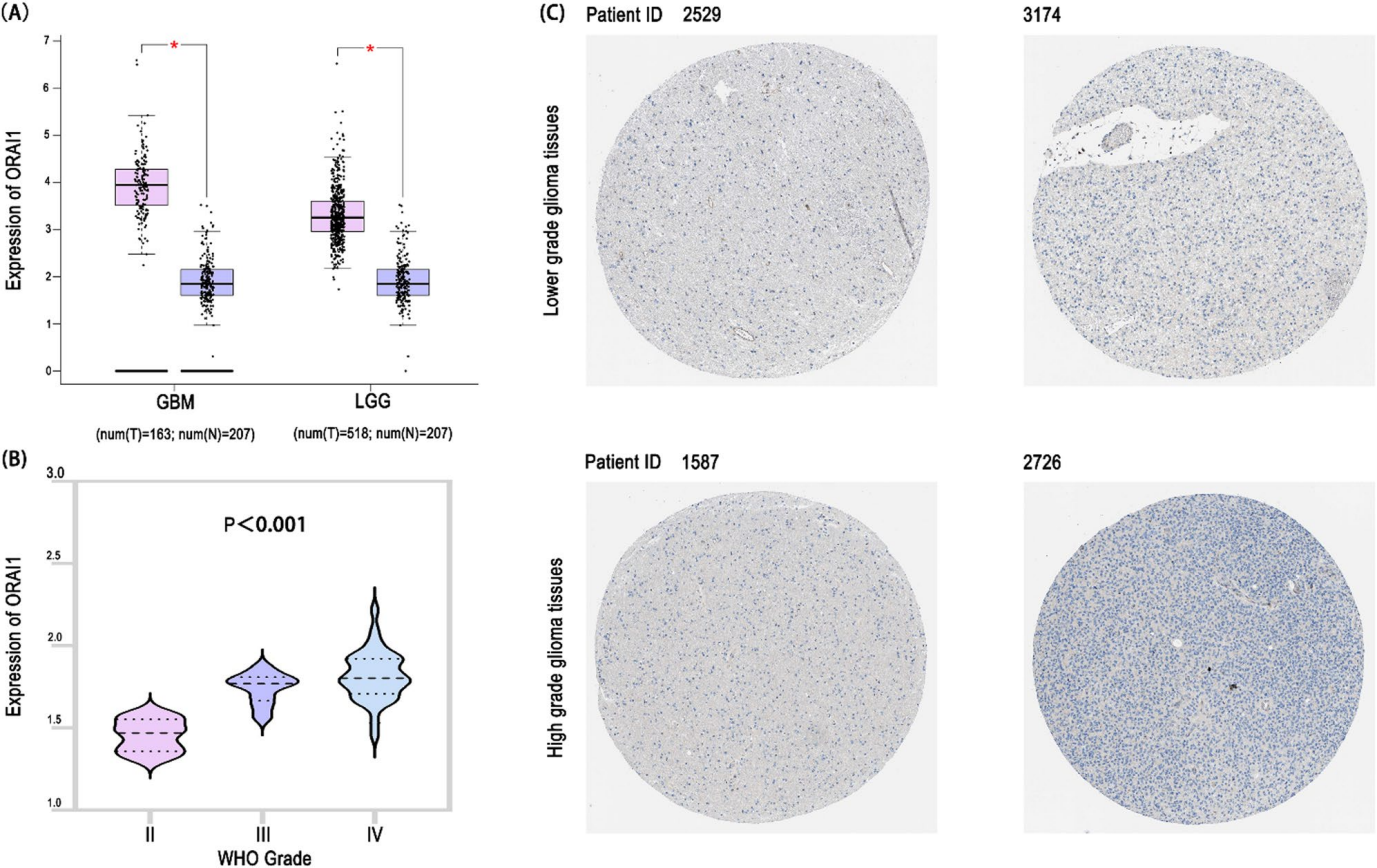
Expression of ORAI1 in glioma tissues and patient plasma. (**A**) GEPIA database analysis shows elevated ORAI1 expression in GBM and LGG tissues compared to normal brain. (**B**) ELISA of plasma from 50 patients reveals increasing ORAI1 levels with higher WHO grades, with significantly higher expression in grade III–IV gliomas (*P* < 0.01). (**C**) Immunohistochemistry images from the HPA database show no detectable ORAI1 protein expression in GBM and LGG tissues.

Analysis of the GEPIA database revealed that ORAI1 mRNA expression was significantly upregulated in both GBM and LGG tissues compared to normal brain tissue (*p* < 0.01) [Fig. 4A]. However, immunohistochemical images from the Human Protein Atlas (HPA) database showed no detectable ORAI1 protein expression in GBM and LGG samples[Fig. 4C], suggesting that ORAI1 protein expression may be suppressed at the tissue level or rapidly transported extracellularly.

Further, in a prospective cohort of 50 glioma patients from our center, plasma ORAI1 levels were measured by ELISA. Results demonstrated a stepwise increase in plasma ORAI1 expression correlating with higher pathological grades (WHO II → III → IV), with levels in grade III and IV patients significantly elevated compared to low-grade gliomas (*p* < 0.01) [Fig. 4B]. These findings suggest that ORAI1 may be released from tumor tissues into peripheral blood via extracellular vesicles (Evs) or active secretion mechanisms.

### Functional annotation of ORAI1 reveals its involvement in cell cycle and ER-related pathways

**Fig. 5:**
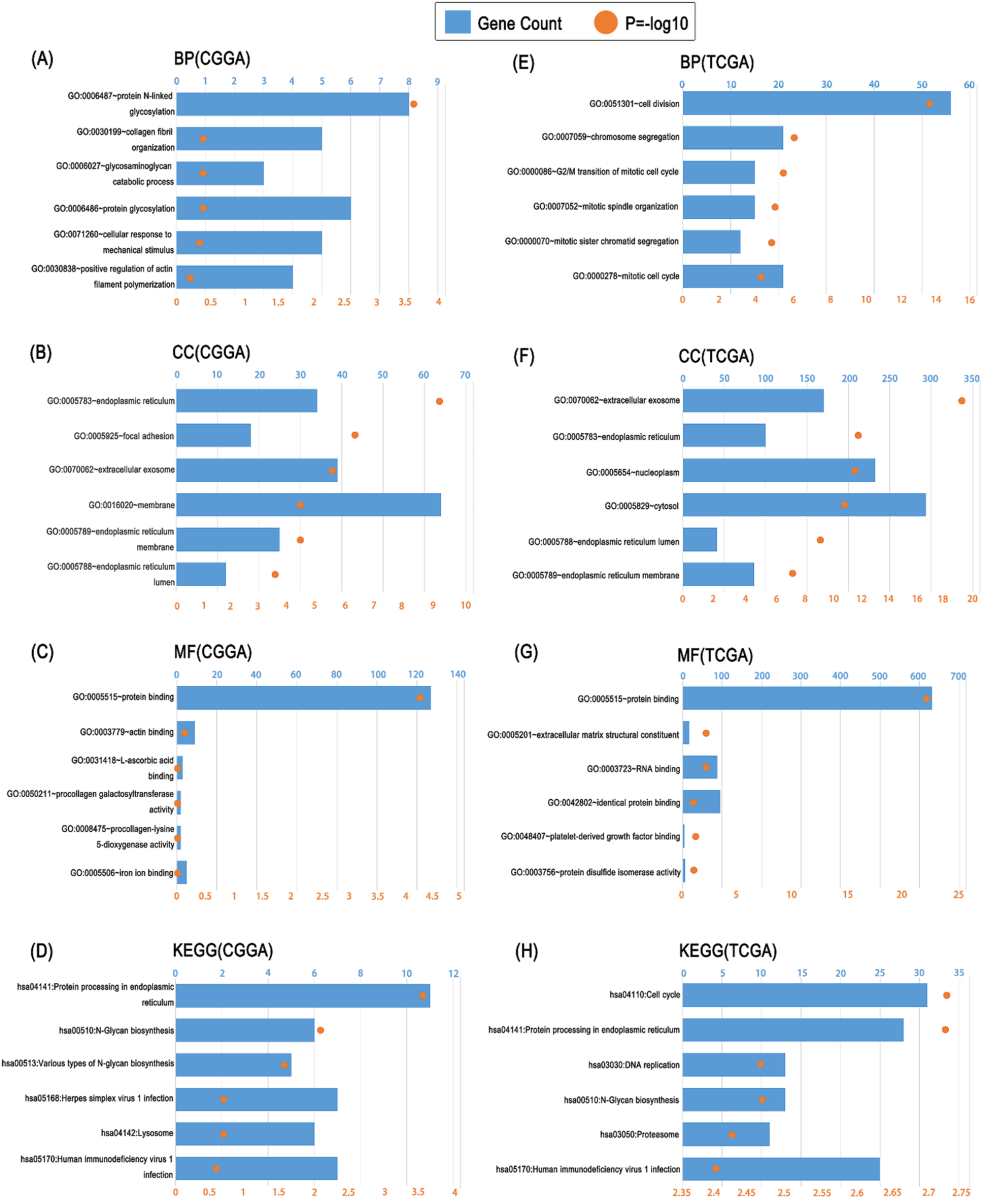
Functional enrichment analysis of ORAI1-associated genes. (**A–D**) GO and KEGG analyses in the CGGA dataset reveal enrichment in biological processes such as protein N-glycosylation (**A**); cellular components including endoplasmic reticulum, focal adhesion, and EVs (**B**); molecular functions mainly involving protein binding (**C**); and pathways related to protein processing in the endoplasmic reticulum and N-glycan biosynthesis (**D**). (**E–H**) Similar enrichment patterns were observed in the TCGA dataset, with cell division as the top biological process (**E**) and consistent cellular components, molecular functions, and pathways (**F–H**).

To investigate the biological functions associated with ORAI1, Pearson correlation analyses (|R| > 0.5, *p* < 0.05) were performed separately on the TCGA and CGGA datasets. GO and KEGG pathway enrichment analyses were conducted based on the correlated gene sets^17–19^. In the CGGA dataset, the biological process most strongly associated with ORAI1 was protein N-glycosylation[Fig. 5A]. The related cellular components included the endoplasmic reticulum, focal adhesions, and EVs[Fig. 5B]. Molecular function enrichment highlighted protein binding[Fig. 5C]. The top enriched pathways involved protein processing in the endoplasmic reticulum and N-glycan biosynthesis[Fig. 5D]. Similarly, in the TCGA dataset, cell division was the biological process most correlated with ORAI1[Fig. 5E], while the enriched cellular components, molecular functions, and pathways closely mirrored those observed in CGGA[Fig. 5F-H]. These findings suggest that ORAI1 has potential as a non-invasive molecular biomarker for glioma, applicable to early diagnosis, prognostic assessment, and therapeutic response prediction.

### Gene set variation analysis identifies ER stress and cell cycle as key pathways associated with ORAI1

**Fig. 6:**
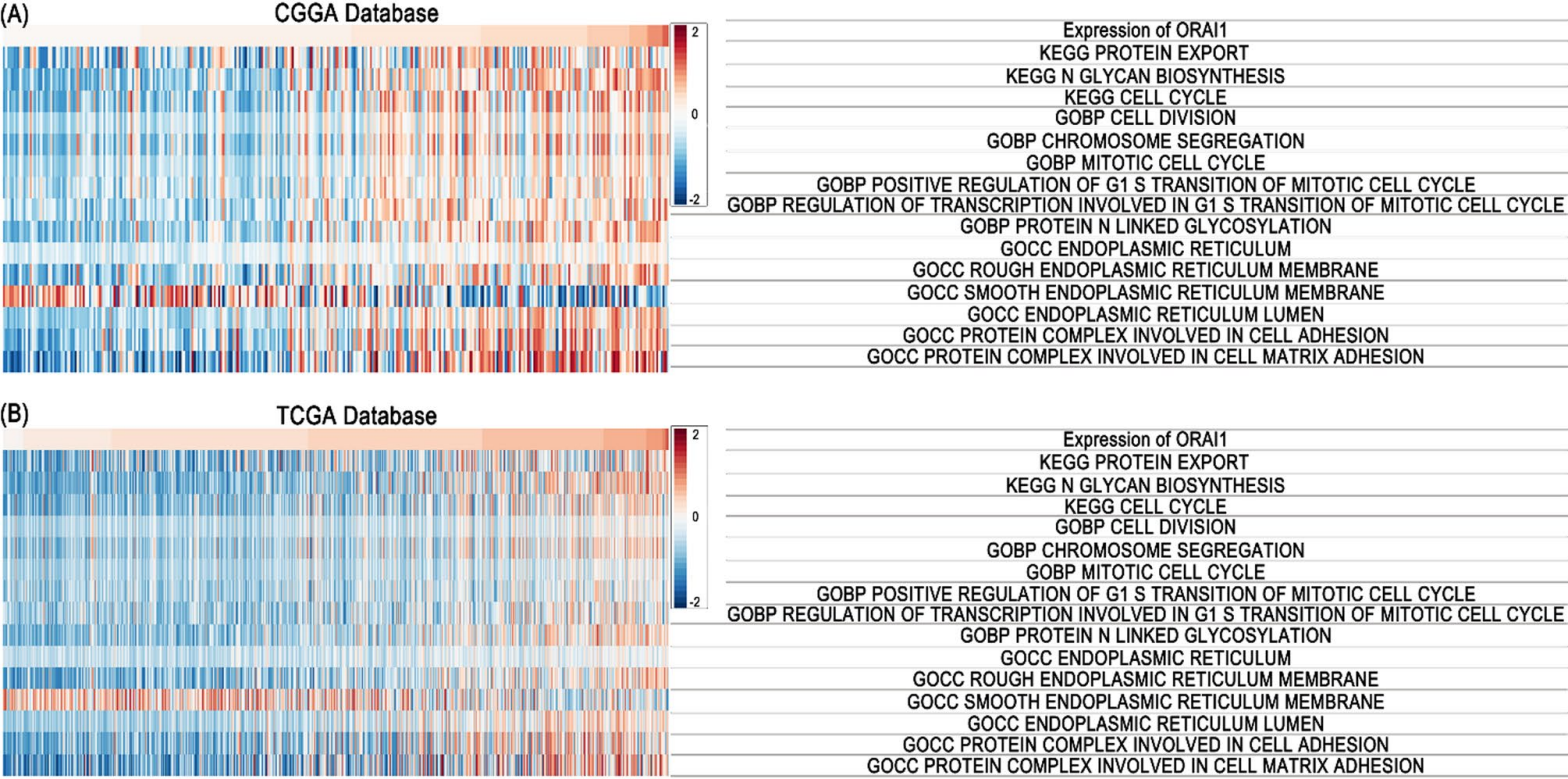
Correlation analysis between ORAI1 expression and functional enrichment scores. (**A**,** B**) Heatmaps depicting ORAI1 expression and associated functional enrichment scores for each patient in the CGGA (**A**) and TCGA (**B**) datasets. Samples are ordered by ascending ORAI1 expression.

To further elucidate the potential functional mechanisms of ORAI1 in glioma, GSVA was performed using data from the TCGA and CGGA cohorts. Correlation analysis between enrichment scores and ORAI1 expression demonstrated a positive association with multiple functional pathways [Table 2], except for the smooth endoplasmic reticulum membrane pathway in the CGGA dataset [Fig. 6A]. These results were validated in the TCGA cohort [Fig. 6B]. Collectively, these findings suggest that ORAI1-mediated cell cycle activation and endoplasmic reticulum remodeling processes may synergistically promote its extracellular release. This warrants further in-depth investigation in the context of clinical blood biomarker screening and validation.

**Table 2:**
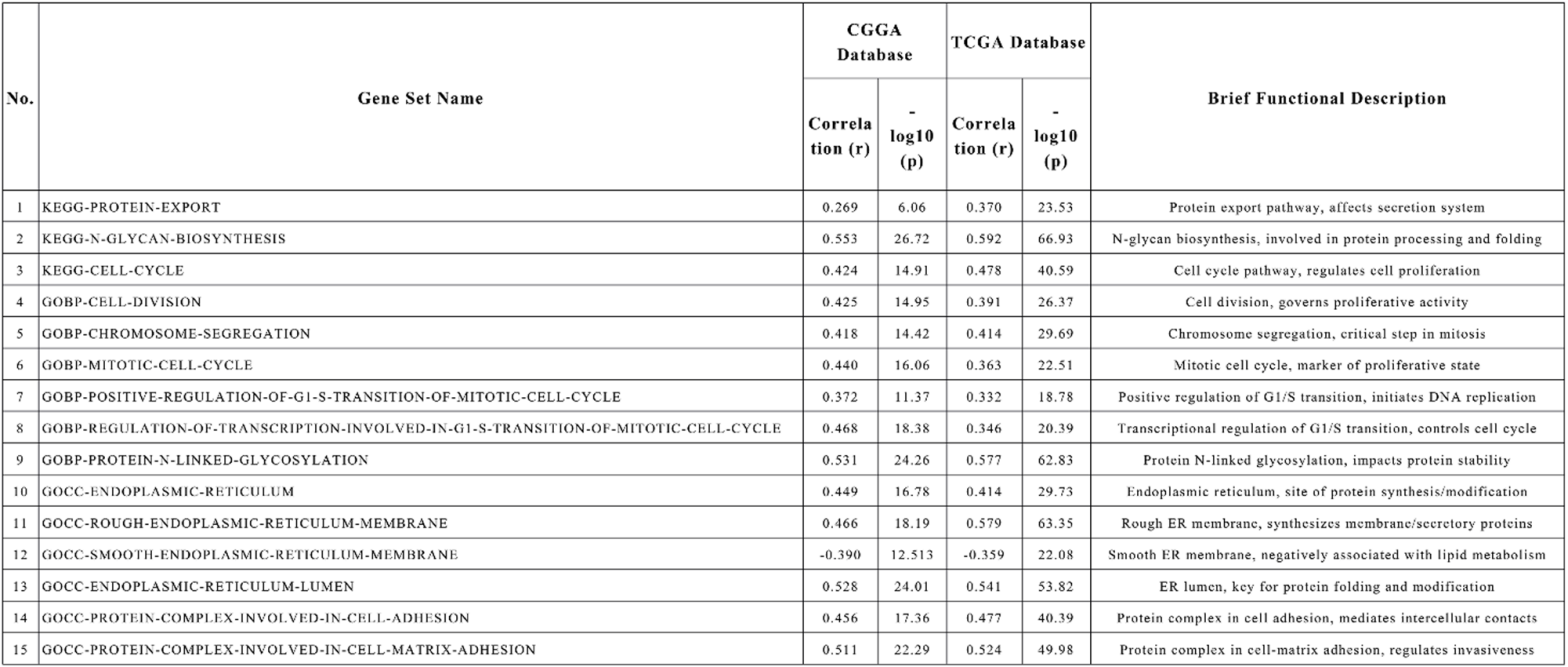
Correlation between ORAI1 expression and functional pathways.

**Table 3:**
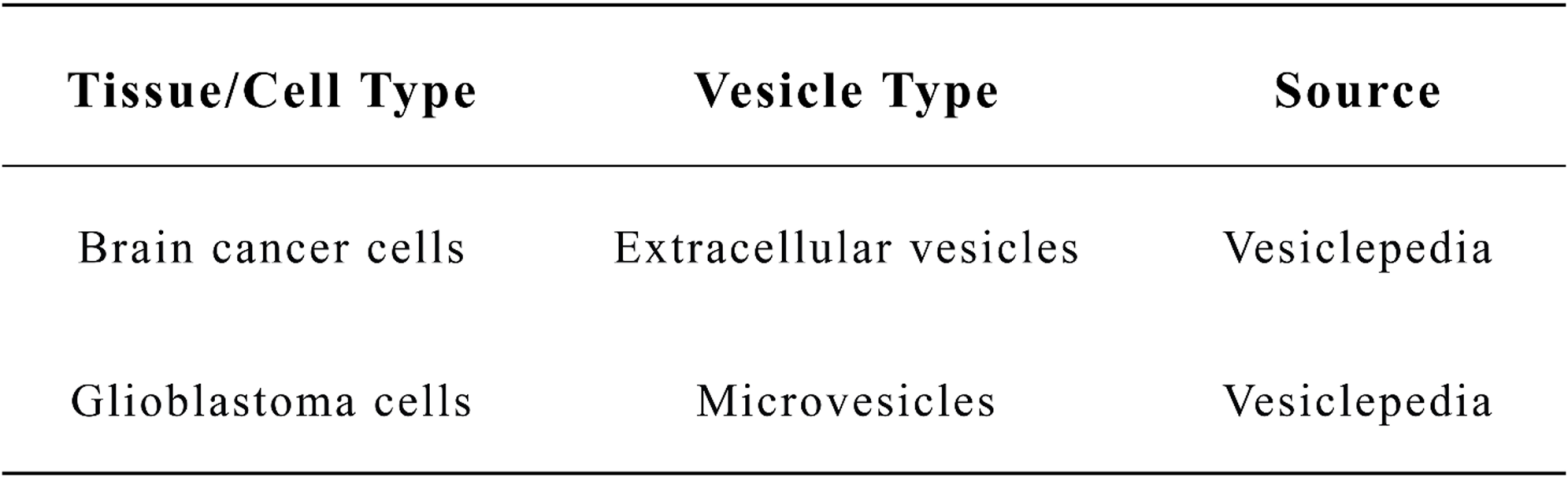
Evidence of ORAI1 expression in EVs from vesiclepedia Database.

### High ORAI1 expression correlates with poor early postoperative symptom scores in glioma patients

**Fig. 7:**
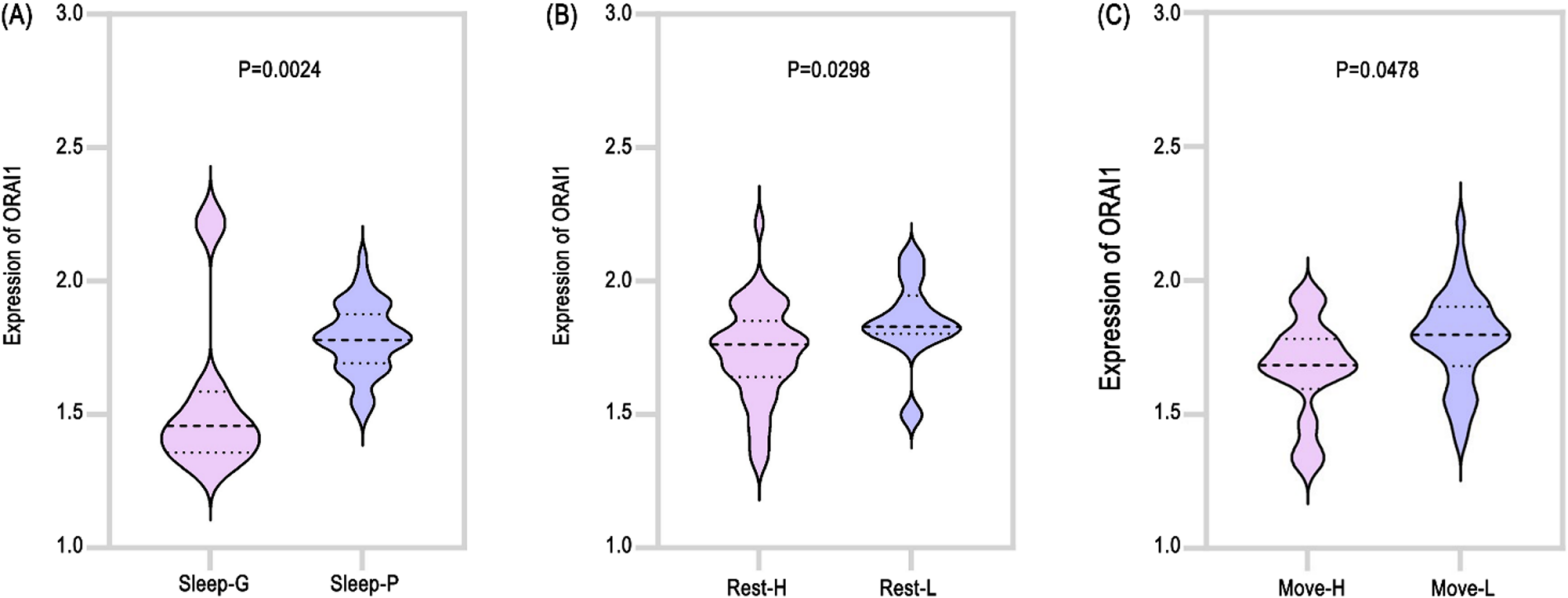
Higher plasma ORAI1 levels correlate with worse postoperative symptoms in a prospective glioma cohort. (**A–C**) Patients with elevated plasma ORAI1 showed significantly poorer postoperative subjective scores, including reduced sleep quality (**A**), increased resting pain (**B**), and increased activity-related pain (**C**) (*P* < 0.01).

To further assess the potential value of ORAI1 in predicting early postoperative recovery status, we analyzed the relationship between plasma ORAI1 levels and multiple short-term symptom scores within 72 h after surgery in a prospective glioma patient cohort from our center. These included sleep quality, activity-related pain scores (NRS-activity pain), and resting pain scores (NRS-resting pain). The analysis demonstrated that patients with poorer sleep quality exhibited higher ORAI1 expression, suggesting its potential predictive significance for early postoperative sleep disturbances (*p* < 0.01) [Fig. 7A]. Additionally, patients reporting higher pain scores both during activity and at rest also showed elevated ORAI1 levels (*p* < 0.01) [Fig. 7B, C], indicating a close association between ORAI1 expression and acute postoperative pain severity. These findings highlight ORAI1 as a promising non-invasive blood-based molecular biomarker for predicting early postoperative recovery, potentially aiding clinical decision-making regarding pain management and early rehabilitation strategies.

## Discussion

Gliomas, particularly GBM, represent one of the most aggressive and lethal forms of primary brain tumors, with poor prognosis and limited treatment response despite advances in surgical techniques, chemotherapy, and radiotherapy^20^. Traditional prognostic systems rely heavily on histological grading and molecular markers such as IDH mutation, 1p19q co-deletion, and MGMT promoter methylation^5^. However, the need for intraoperative or postoperative tissue limits their use in real-time monitoring or early diagnosis. In recent years, there has been growing interest in discovering blood-based biomarkers that can noninvasively reflect tumor burden and biological behavior^21^. In this context, ORAI1, a key calcium channel regulator implicated in calcium influx and ER stress, has emerged as a potential candidate in several malignancies^22–24^, yet its role in gliomas remains largely unexplored and requires further investigation.

One of the key clinical challenges in neuro-oncology is identifying biomarkers that not only reflect tumor progression and prognosis but also correlate with patient-centered outcomes such as QoL^25^. While numerous tissue-based markers have been identified, their predictive value is often limited by sample accessibility and tumor heterogeneity. Blood-based indicators, particularly those detectable in EVs or plasma, offer significant advantages in noninvasive monitoring and individualized treatment planning^26,27^. However, few biomarkers have demonstrated consistent value across multiple data platforms and clinical endpoints. Whether a single molecule could simultaneously inform tumor grade, prognosis, and patient well-being remains an open question.

In this study, we investigated the biological role and clinical value of ORAI1 in glioma using a combination of bioinformatic analyses and clinical cohort validation. Functional annotation based on co-expression profiles in the CGGA and TCGA datasets revealed that ORAI1 is significantly involved in ER-related pathways such as protein N-glycosylation and protein processing in the endoplasmic reticulum, as well as cell cycle regulation including mitotic progression and chromosome segregation. GSVA further suggested its involvement in ER stress and proliferative signaling pathways, including significant enrichment in KEGG_CELL_CYCLE and GOBP_MITOTIC_CELL_CYCLE. Notably, all enriched pathways were positively correlated with ORAI1 expression except for GOCC_SMOOTH_ENDOPLASMIC_RETICULUM_MEMBRANE, which showed a consistent inverse correlation in both datasets, potentially indicating a specific ER remodeling state associated with aggressive tumor behavior.

At the tissue level, our findings presented a compelling paradox. While GEPIA analysis showed elevated ORAI1 mRNA expression in tumor tissues compared to normal controls, immunohistochemistry from the Human Protein Atlas (HPA) revealed little to no detectable protein in both LGG and GBM samples. This discrepancy led us to examine the peripheral blood expression of ORAI1 in a prospective cohort of 50 glioma patients. Strikingly, ORAI1 levels in plasma increased significantly with ascending WHO grades, with highest concentrations observed in grade III–IV tumors. This tissue–plasma dissociation pattern may indicate active secretion or vesicle-mediated export of ORAI1 from tumor cells, possibly driven by ER stress or calcium signaling activation. Previous studies have shown that ORAI1-mediated Ca²⁺ influx can promote vesicle release and exocytosis in breast and colon cancer models^28–30^, supporting this hypothesis as a tentative model in glioma.

Mechanistically, we propose that upregulated ORAI1 expression may induce calcium imbalance and ER stress, which could in turn disrupt the integrity of the smooth endoplasmic reticulum (SER) membrane, potentially leading to enhanced extracellular vesicle formation and secretion. The negative correlation between ORAI1 and GOCC_SMOOTH_ENDOPLASMIC_RETICULUM_MEMBRANE further supports this model. This ER remodeling may facilitate the transport of ORAI1 into the peripheral circulation via vesicle packaging, thereby increasing its detectability in plasma while reducing its tissue retention. This model is consistent with recent evidence suggesting that ER stress may contribute to the packaging of tumor-derived proteins into EVs, facilitating distant communication and immune modulation^31,32^. Thus, ORAI1 may not only serve as a passive marker but also actively participate in reshaping the tumor microenvironment. Moreover, previous work has shown that ORAI1, as a Ca²⁺ channel protein, might regulate the Ca²⁺-dependent release of PD-L1–positive EVs^33^, further supporting its putative mechanistic involvement in EV-mediated secretion. In line with our findings, data from the Vesiclepedia database indicate that ORAI1 is present within extracellular vesicles, including both microvesicles and exosomes. This observation suggests that the presence of ORAI1 in circulation is more likely due to selective vesicular secretion rather than passive leakage[Table 3]. Pan-cancer analyses have recently suggested that mesenchymal stem cell (MSC) signatures, which interact closely with tumor microenvironment components, serve as independent prognostic markers in glioma and other cancers, highlighting the critical influence of stromal-immune crosstalk on clinical outcomes^34^.

Beyond molecular and histological markers, one of the most novel aspects of our study lies in the correlation between ORAI1 expression and early postoperative recovery status. High ORAI1 expression was associated with significantly poorer sleep quality and higher NRS scores for both resting and activity-related pain. These associations suggest a link between tumor-derived ORAI1 signaling and neural or inflammatory processes affecting nociception and sleep regulation. While the exact pathways remain to be elucidated, calcium dysregulation, neuroinflammation, and ER stress are all known contributors to sleep disorders and pain sensitivity^35,36^. By connecting tumor biology with clinical outcomes, ORAI1 demonstrates significant translational potential as a dual-function biomarker for diagnosis and prognosis. Given the inherent sub-clonal heterogeneity and complex tumor microenvironment in glioblastoma, emerging evidence suggests that personalized approaches including gene-targeted therapies may offer improved efficacy. In this context, non-invasive biomarkers such as ORAI1 could provide critical guidance for patient stratification and treatment planning^37^.

Importantly, our ROC curve analyses further validated the clinical utility of ORAI1 as a predictive biomarker. In both CGGA and TCGA cohorts, ORAI1 outperformed classical markers such as 1p19q co-deletion and MGMT promoter methylation in predicting WHO tumor grade, with AUC values of 0.811 and 0.751, respectively. Given that ORAI1 is detectable in peripheral blood and correlates well with tumor grade and prognosis, it holds considerable promise as a noninvasive, dynamic biomarker for early diagnosis, tumor classification, and longitudinal disease monitoring. Moreover, its added association with QoL parameters positions it as a potential tool for postoperative management and rehabilitation planning. ORAI1 provides broader translational accessibility compared to tissue- or mutation-restricted biomarkers.

Nonetheless, this study has several limitations. First, the mechanistic role of ORAI1 was inferred solely from bioinformatic analyses without experimental validation, leaving its specific function in tumor–host interactions unclear. Second, the prospective clinical cohort for QoL validation was relatively small (*n* = 50), and postoperative assessments were limited to a single early time point, restricting insight into longitudinal changes. Third, potential confounding factors—such as patients’ psychological status, tumor size, surgical trauma, and operative duration—were not fully controlled, which may affect QoL outcomes. Fourth, the absence of independent multicenter validation limits the generalizability of our findings. Future studies with larger, longitudinal cohorts and mechanistic experiments are warranted to address these limitations and further elucidate ORAI1’s clinical and biological roles.

## Conclusion

In summary, our study provides the first comprehensive evidence that ORAI1 serves as a clinically relevant biomarker in glioma, showing broad potential in tumor classification, survival prediction, and evaluation of early postoperative recovery, which may further contribute to the prediction of long-term quality of life outcomes.

## Supplementary Information

Below is the link to the electronic supplementary material.

Supplementary Material 1

Supplementary Material 2

Supplementary Material 3

Supplementary Material 4

Supplementary Material 5

## Data Availability

The datasets analyzed during the current study are publicly available in the CGGA (http://www.cgga.org.cn/), TCGA (https://portal.gdc.cancer.gov/), Gene Ontology (GO), Kyoto Encyclopedia of Genes and Genomes (KEGG), Molecular Function (MF), Human Protein Atlas (HPA; https://www.proteinatlas.org/), and Vesiclepedia (http://microvesicles.org/) repositories. The clinical datasets generated and/or analyzed during the current study are available from the corresponding author on reasonable request.

## Abbreviations

QoL: Quality of life
CGGA: Chinese glioma genome atlas
TCGA: The cancer genome atlas
GBM: Glioblastom
LGG: Lower-grade glioma
SOCE: Store-operated calcium entry
IRB: Institutional review board
IDH: Isocitrate dehydrogenase
MGMT: O6-methylguanine-DNA methyltransferase
GO: Gene ontology
KEGG: Kyoto encyclopedia of genes and genomes
GSVA: Gene set variation analysis
ROC: Receiver operating characteristic
NRS: Numeric rating scale
PSQI: Pittsburgh sleep quality index
EVs: Extracellular vesicles
AUC: Area under the curve
MSC: Mesenchymal stem cell
