# Supplementary material for: Plasma biomarker ORAI1 as a dual prognostic value for survival and postoperative quality of life in glioma patients
Source: Sci Rep. 2025 Dec 30;16:4198. doi: 10.1038/s41598-025-34228-4 (PMC12859135; doi:10.1038/s41598-025-34228-4)
Supplement: Supplementary file 1 — Supplementary Material 1 [file 41598_2025_34228_MOESM1_ESM.doc]

**Highlights**

1. The inverse association between Orai1 and SER membrane suggests a novel mechanism of biomarker release through ER-membrane destabilization and exosomal export, supporting its clinical utility as a noninvasive prognostic tool.
2. Plasma Orai1 is identified as a novel, noninvasive dual biomarker that independently predicts both overall survival and postoperative quality of life in glioma patients.
3. Orai1 shows a tissue–blood expression dissociation pattern, possibly due to ER remodeling and exosomal secretion, suggesting a novel mechanism of biomarker release.
4. The study integrates large-scale multi-ethnic transcriptomic datasets with a prospective Chinese plasma cohort, enhancing translational reliability and clinical relevance.
