# Supplementary figures and images for: Plasma biomarker ORAI1 as a dual prognostic value for survival and postoperative quality of life in glioma patients
Source: Sci Rep. 2025 Dec 30;16:4198. doi: 10.1038/s41598-025-34228-4 (PMC12859135; doi:10.1038/s41598-025-34228-4)

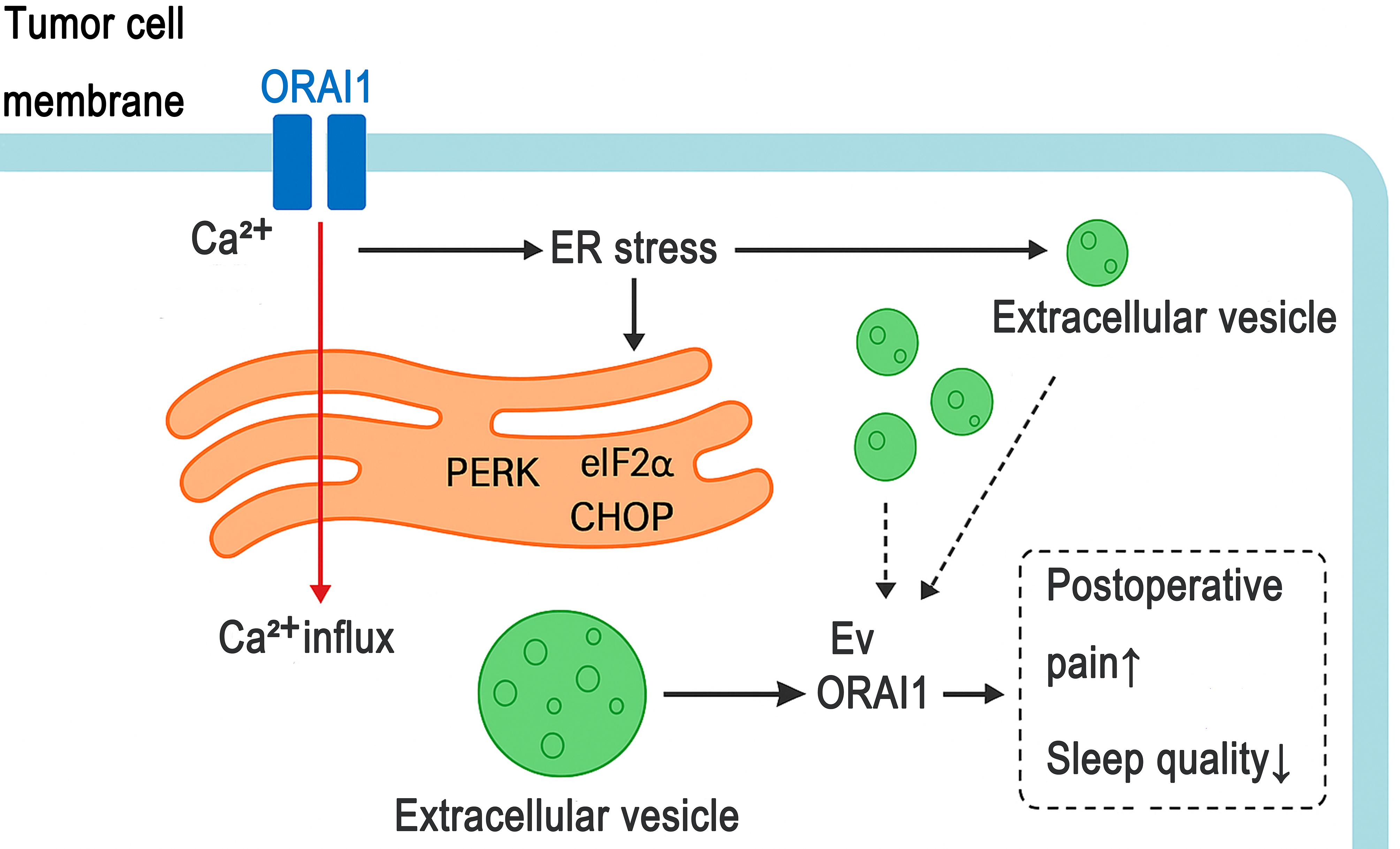

Supplement: Supplementary file 2 — Supplementary Material 2 [file 41598_2025_34228_MOESM2_ESM.tif]

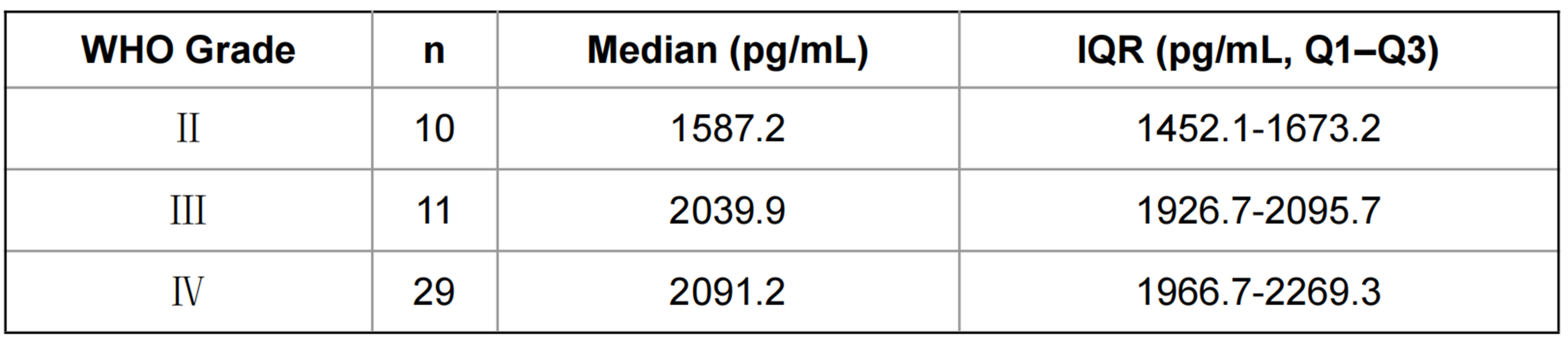

Supplement: Supplementary file 3 — Supplementary Material 3 [file 41598_2025_34228_MOESM3_ESM.tif]
