# Supplementary material for: Plasma biomarker ORAI1 as a dual prognostic value for survival and postoperative quality of life in glioma patients
Source: Sci Rep. 2025 Dec 30;16:4198. doi: 10.1038/s41598-025-34228-4 (PMC12859135; doi:10.1038/s41598-025-34228-4)
Supplement: Supplementary file 4 — Supplementary Material 4 [file 41598_2025_34228_MOESM4_ESM.doc]

Supplementary Table S1.

Median and interquartile range (IQR) of plasma Orai1 concentrations stratified by WHO glioma grades


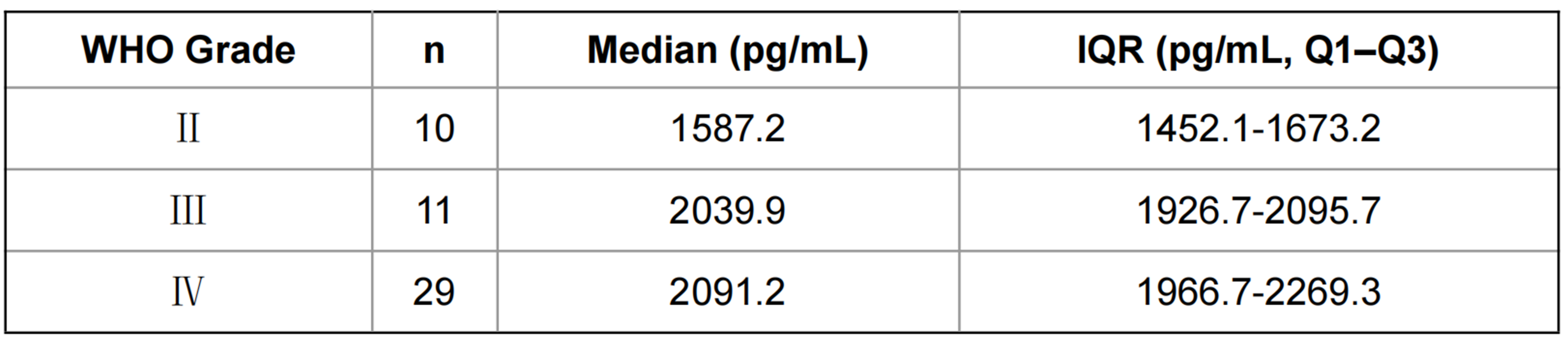


Legend:

Values are presented as median (interquartile range). IQR indicates interquartile range. Plasma Orai1 concentrations were measured by ELISA.
