# Supplementary material for: Plasma biomarker ORAI1 as a dual prognostic value for survival and postoperative quality of life in glioma patients
Source: Sci Rep. 2025 Dec 30;16:4198. doi: 10.1038/s41598-025-34228-4 (PMC12859135; doi:10.1038/s41598-025-34228-4)
Supplement: Supplementary file 5 — Supplementary Material 5 [file 41598_2025_34228_MOESM5_ESM.doc]

Supplementary FIG S1.

Orai1-mediated Ca²⁺ influx triggers ER stress and extracellular vesicle (EV) secretion, contributing to postoperative pain and sleep disturbance.


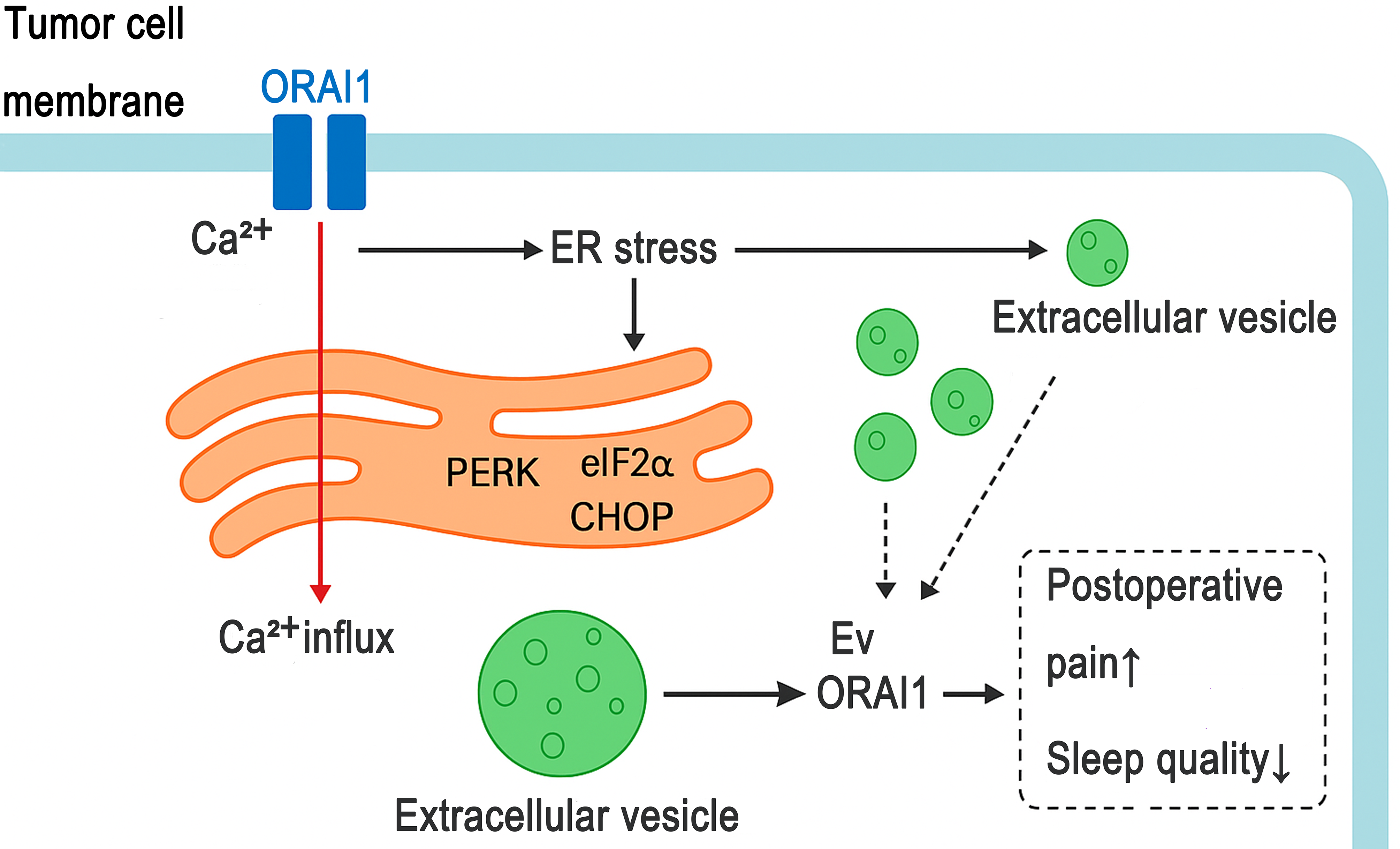


Legend:

Orai1, a plasma membrane calcium channel, mediates Ca²⁺influx and induces ER stress via the PERK–eIF2α–CHOP pathway, leading to enhanced secretion of extracellular vesicles (EVs) carrying Orai1 and/or related signals. These EVs modulate downstream Orai1-dependent pathways and are implicated in increased postoperative pain and impaired sleep. Solid arrows (→) indicate direct effects, dashed arrows (---→) indicate indirect or hypothetical pathways, and red arrows highlight the central role of Ca²⁺influx.
